# From the Imaginary to Theory of the Gaze in Lacan

**DOI:** 10.3389/fpsyg.2021.578277

**Published:** 2021-03-30

**Authors:** Carmelo Licitra Rosa, Carla Antonucci, Alberto Siracusano, Diego Centonze

**Affiliations:** ^1^IRCCS Neuromed, Pozzilli, Italy; ^2^Department of Systems Medicine, Tor Vergata University, Rome, Italy

**Keywords:** gaze, Lacan, *object a*, mirror stage (lacan), imaginary, phallus, scopic drive, remotepsychoanalytic treatment

## Abstract

To understand Lacan’s thinking process on vision, the entirety of his teaching must be taken into consideration. Until the 60s, the visual field is the imaginary, the constitutive principle of reality in its phenomenal giving to the experience of a subject. This register is the opposite of the field of the word with the L schema and, subsequently, as subordinated to the symbolic system according to the model of the optical schema of the inverted flower vase of Bouasse. It is only with the 1964 seminar that Lacan makes a daring turnaround through which the visual becomes a sign of the emergence of a real that is irreducible to both reality and the mediation of the subject of knowledge. The split that separates reality and the real is reproduced in Lacan within the visual field, which is, on the one hand, the cardinal principle of the consistency of the experience of reality (as imaginary), and on the other, it is an element of irreducibility to reality (as object gaze). This produces a cascade of consequences: first of all, the modification of the presentation of the mirror stage. Unlike the voice, which through prosody, tone, and volume, finds some strips with which anchor itself imaginatively to reality, the gaze, invisible and elusive, escapes the imaginary grasp. Captured in myths, it reveals its power and ability to annihilate—as in the myth of Medusa’s gaze—or to make people fall in love but only with a narcissistic love that leads inexorably to death as in the myth of Narcissus. The gaze is elusive because the subject is dependent on it in the field of desire. Like the voice, it is about the desire on which the subject is supported; it is one of the objects on which the phantom depends. In our opinion, thanks to this characteristic, the gaze object can make remote psychoanalytic treatment possible through easily accessible videoconferencing tools and, at the same time, create new conditions within it that should be carefully evaluated to understand its implications in the session itself.

## Introduction: For a Theory of the Imaginary Correlated to the Function of the Gaze

The imaginary in Lacan’s teaching underwent a number of revisions and changes over the years. The imaginary order is the first encounter of the subject with the world in which the ego searches for itself an imagined identification: In the mirror stage, prior to the formation of the discourse of the demand, the imaginary structures the identification of the self based on an interaction between the self and an external other, which is symbiotically attached to the narcissistic body.

Starting from 1949 with the L schema, the imaginary is modulated by Lacan as a register opposite to the field of the speech (Lacan, 2006, [1949]). In this opposition, the imaginary is basically superimposed on the field of language, intended as a fundamental opposition to the function of the speech. Subsequently, it was subordinated to the symbolic system according to the model of the optical schema of the inverted flower vase of Bouasse (Lacan, 2006, [1960a]).

Later, with the essay “Subversion of the Subject and Dialectic of Desire in the Freudian Unconscious” Lacan (2006, [1960b]), followed in 1962 by *The Seminar of Jacques Lacan, Book X, Anxiety* Lacan (2014, [1962]), the reference model for a crucial reinterpretation of the imaginary is the transfer of the libido from the body to the object. A part of this libido would, however, remain almost preserved from this transfer in such a way that—like the mythological Achilles heel—this body part, not immersed, would end up concentrating in itself an irreducible nucleus of autoeroticism. Where would this libido be concentrating so as to remain preserved from immersion or transfusion? In the penile appendix, answers Lacan.

Now, comparing the tip position and shape of this appendix to the whole form nominates it to become the object of a phantom of caducity—caducity that would be accomplished in the exclusion of the appendix itself from the mirror image (with a hole resulting in it as a consequence) and the prototype to which this is elevated in the world of objects. It is in this sense that the statement that the phallus, i.e., the image of the penis, is made negative in the place where it should be found in the mirror image should be understood.

We can then say that, in the constitution of the ego insofar as it is narcissistic, there is an intrinsic limit to the libidinal investment from the body to the image of the other: The libido from the body, which would be its source, does not flow entirely on the mirror image. Therefore, a blind area remains in the image, called -φ.

This is why the male, at the sight of the absence of the penis in the female, can imagine what could happen to him, and this is also why the female, at the sight of this presence in the male, imagines what she presumes to have lost: This yet to happen on one side and this already happened on the other side bring both back to the same -φ in the image. If, on the male side, there is a fear of the loss that could be suffered, on the female side, there is a nostalgia for this same loss already suffered. The crucial point to grasp is that, for both in the image of their own body, the phallus is something to be conceived as less, as a blind spot, as a free zone, preserved.

It can also be said that the phallus is a libidinal reserve not represented in the image but implicated in that which is ousted from the image insofar as it traces the edges of its own absence. In place of this hole, which we have specified as -φ, is where the object is located because of desire: This allows us to understand why the *object a* can join the phallus only when this phallus is a little off stage. Because phallic jouissance is interdicted, the surplus-jouissance function of the drive objects comes in its place as a substitute.

## The Imaginary and the Gaze

If one follows carefully the texts of this period of Lacan’s teaching (the decade between 1960 and 1970), one will be struck by the insistence with which his speech hammers on the theme of the gaze.

Thus, we find, first of all, the return to the dream “Father, can’t you see I’m burning?” (Freud, 1900), then the reference to a poem by Aragon (1963; the other is my reflection but without a gaze), then the introduction to a posthumous book by Merleau-Ponty (1968) titled *The Visible and the Invisible*, then the reference to Caillois (1960) and his research on the *ocelli* of mimicry, and finally the analysis of the gesture of the painter—like Pollock—who throws something to see to our hungry gaze. To close this overview, we find the analyst defined as the one whose gaze is hypnotized by the analysand in a sort of inverted hypnosis.

What is behind all this insistence?

Lacan presents the list of the drives—four drives: making oneself seen, heard, sucked, and shitted (Lacan, 1988, [1964a]). It is immediately evident that, every time, whatever the form of the drive, the object of the drive is always placed in the field of the other.

Now, with regard to the scopic drive, Lacan points out that Freud himself remarked that it was not homologous to the other three, that is, that it has a structure of its own. The gaze is, in fact, the most suitable term to grasp the proper function of the *object a* as cause of desire insofar as it presents itself rightly in the field of the mirage of the narcissistic function as an elusive object, unspeakably exterior, beyond the image of the narcissistic object that I can see in the vision.

Here, we must recall the famous framework proposed by Freud (1921) in *Group Psychology and Analysis of Ego* (1921) in which the beyond the object is well represented with respect to the field of identification that founded narcissism. In this schema, we find clearly indicated the places of the ideal of the ego (I), of the narcissistic object (II), of the ego (III) and then, outside like a vanishing point, that of the object beyond. Moreover, in this same schema, the orientation of the vectors shows very well the possible confusion that the subject can try to accomplish by overturning the *object a* on the idealizing identification to evade castration.

Let us just note how, of all the drive objects, Lacan chooses the scopic object, the gaze, to show the relationship of conjunction/disjunction between the drive and the imaginary body and, from here, to assert that the gaze, more than any other drive object, imposes a modification in the presentation of the imaginary.

The field of the visible was introduced by Lacan with the mirror stage: This is the point of origin of any discourse on the visible. Seeing the image of the other’s body, I anticipate the mastery of my body and totalize my mirror image; this is at the origin of the narcissistic satisfaction of oneself.

But—and here is the novelty—what was I before this origin? Answer: a being looked at, looked everywhere, exposed. I am originally in the show even before I constitute the other as the object of this show.

The publication of Merleau-Ponty’s posthumous book *The Visible and the Invisible* offers Lacan the opportunity to insist on this moment prior to the specular origin of visibility. In fact, Merleau-Ponty says that the one who sees is absorbed in what he sees, in such a way, as a result, there would, therefore, be a fundamental narcissism of every vision and that, for this same reason, vision that he who sees exercises at the same time he is subjected to through the things themselves. Just as many painters have glimpsed, I feel myself looked at by things, my activity is at the same time passivity, something constitutes the second and deepest sense of narcissism. This general visibility of the sensitive as in itself, this innate anonymity of myself is what we may call flesh although, in the philosophical tradition, there is no suitable term to designate this point. So far, Merleau-Ponty, now Lacan.

Now, turning to Lacan, he moves from here to emphasize how this primordial reality that is on this side of the mirror stage is neither reduced nor erased by it, but remaining on this side of the image, it questions the mirage of narcissistic satisfaction of the image. However, if I am a being looked at, what would the gaze be then? It cannot be the eye, which is the organ of vision; it must be something in its own right, the peculiarity of which can be grasped by articulating the fundamental relationship between gaze and stain, given that, in the world, there is something to look at before there is a sight to see it. In this way, the ocellus of animal mimicry is the essential presupposition of the fact that a subject can then, at a later time, see it and remain enchanted by it: in other words, the fascination with the stain is antecedent to the sight that discovers it.

The gaze, therefore, is not the eye, and if the ocelli impress it, it is not because they resemble eyes—this is the contribution of Roger Caillois valued by Lacan (1988, [1964b])—but on the contrary, the eyes are able to arouse a sense of intimidation because they resemble ocelli.

The magnetism of the ocelli would, therefore, seem to descend from the fixed and brilliant circular shape as a factor of fascination.

Caillois, in fact, finds mimicry in its three fundamental manifestations of disguise, camouflage, and intimidation and, in this way, enhances the conditions of a fascination prior to the actual presence of the other’s eye.

Now, the key point to be grasped is that, of this dimension proper to the animal kingdom, the human being carries out an important manipulation, transforming it into a given to see, mask, and at the same time reduplicate himself. Thus, the gesture of the painter deposits stains on the canvas so that the spectator can deposit its eye on it. Do you want to watch? Well, here is this to see: the little blues, the little whites of Cezanne.

Therefore, we can see that the gaze in the field of the other is what appears to me as a stain. Now this cleft between the gaze and the eye is like a missed appointment, therefore, a real effect: What I look at is never what I want to see in the other, and of the other, because what is presented is a veil, something beyond which I ask to see.

But the same cleft is found by reversing the order of this dialectic. In fact, because I have been seen from all sides since the beginning, here, in my turn, I enter the game of letting myself be looked at by showing off provocatively: I stain, stain that I show on the painting, that I am myself with my own image and this to establish again a lack of the field of the other—you do not look at me from whence I see you.

In both cases, from this cleft between the subject and the other, castration arises as a phallic lack: acknowledgment of the impossibility to dominate the point in the other from where what the subject to be seen is looked at.

Here is the story told by Lacan, a biographical episode dating back to when he was 20 years old (Lacan, 1988, [1964a]). A young Parisian bourgeois wanted to participate 1 day in the hard work of a family of fishermen from Brittany: a noble intention in a time when Maoism was not yet born. Just before pulling the nets up, the young fisherman points out to Lacan a small box of sardines floating on the surface of the water and glistening in the sun. The young fisherman says to Lacan: Do you see that box, do you see it? Well, it doesn’t see you.

Forty years later, Lacan returns to this episode. Why does the young fisherman speak like that? Because evidently this shimmering box does not see you but looks at you as if to bring out the discordant note of the situation: What are you doing, young bourgeois, among these people whom you wanted to sneak between? This means exactly that I am implicated, called upon in that situation, and I cannot wash my hands of it.

This bright spot outside is what I am just like a stain in the painting of this world as it has been looked at since my birth. I am caught there in space and inscribed in a mirror function to the extent that each subject is immediately inserted spatially while remaining other than what it is.

This gaze raises questions with Lacan. This young city intellectual stains the context of workers who laboriously earned their daily living. Here, we find the threefold function of disguise, camouflage, and intimidation: the camouflage of the young intellectual in the situation of the fishermen does not impress the young fisherman, who expresses it by pointing to the tin of sardines. With its shimmers, which recall the glitter of the agalma, this little box shows Lacan what he is missing, that is, the gaze point whence the young fisherman looks at him. What does this cleft between the eye and the gaze refer to? Answer: to a hole in the image of the other, to a -φ, exactly where the *object a* places itself.

## A Third Edition of the Imaginary?

The introduction of the gaze as *object a* in the field of the other produces a cascade of consequences, first of all, the modification of the presentation of the mirror stage.

We have seen that the scopic object, more than any other drive object, has a very close link to narcissism: This is what the mirror phase has taught us since 1936. This modification took place in 1966, precisely in the period in which the publication of the Écrits took place.

Through five prefaces—titled Ouverture Lacan (2006, [1966e]), On My Antecedents Lacan (2006, [1966c]), On the Subject Who Is Finally in Question Lacan (2006, [1966d]), On A Purpose Lacan (2006, [1966a]), and On an Ex Post Facto Syllabary Lacan (2006, [1966b])—and mixed with previous texts recovered and ordered to form the volume of the Écrits (which is precisely the collection of writings prior to 1966), Lacan revises his entry into psychoanalysis and its main inventions.

He explains the delay by justifying it with pedagogical reasons of exposure to an audience not so ready to accept its new ideas, but above all, it projects a new light on his previous writings, in particular, the preface of *On My Antecedents* from a new presentation of the mirror stage, and accounts for the long journey covered by its invention: before 1953, after 1953, after 1966.

Before 1953, Lacan presents an imaginary in a pure state, in which the primacy of vision prevails, thanks to which the child forms its ego from the corporeal image of the other as seen in its totality. The data that comes to Lacan from the Gestalt (Kohler and Buhler) and from the animal ethology (Chauvin, Harrison) and also from the phenomenology, supports him to assert the incidence of envy (whose root is the Latin verb *videre*) as the foundation of the brotherly complex and aggressiveness.

The human space originally has a geometric, kaleidoscopic structure: the beautiful form as I see it in the field of the other fascinates me and structures my own field; then, later, by reflection the beautiful form will be projected in the field of the other in comparison in competition and the warlike conquest of other people’s space. Therefore, the image of the unified form predominates insofar as it is primitive, the geometry of the sack that determines full totalities, surfaces seen as the frontiers of a volume, in support of the hypothesis of a substance that would be its permanent unity below the changing appearances. Finally, this space of the ego determines thought as intuition, which orders its world as a sphere, delimiting the grasp of the concept in its two dimensions of extension and understanding, which are then the same along which it stretches out and closes its hand.

At all levels, therefore—material objects, limits of territory, theory of thought, organization of political and religious action—it reigns a bodily imagination shaped according to the geometry of the ego and its image: here, mastery, unity, and stability find their Euclidean foundation.

The second stage of this journey begins in 1953 when Lacan doubles the first alienation of the image pair of one’s own body/image of the other with a second alienation, symbolic, according to which the unconscious of the subject is the discourse of the other.

However, it is not a simple doubling as it would have been if each of the two relations had continued to function autonomously: in fact, the symbolic determines the imaginary and, for this same reason, makes it impure, not absolute, but tied and subordinate.

To illustrate this link, Lacan used Bouasse’s optical diagram of the inverted flower vase between 1953 and 1960. The diagram is correctly called optical because it is the point of support of a geometry of straight lines that supports the path of reflected rays on two combined mirrors. It is a metaphor, optical in nature, to introduce the symbolic in the imaginary because the ideal of the ego of the second Freudian topic determines the ideal ego. This means that Lacan is reworking the process of Freudian identification in a new light: this recovery is necessary to solve the problem that has remained suspended since the case of Aimée and the stage of the mirror, the problem of aggressiveness. In fact, the dual imaginary relationship is a relationship of exclusion: either I or the other; either I kill the other to break this unbearable image, or he kills me by tearing me away from myself. Would there be an alternative solution to this perpetual oscillation between the ego and the ideal ego? The answer is that in fact there is the place of the other, where the signifier is placed. The child in the mirror remains dissatisfied and awaits a testimony, a sign from the one who occupies the place of the other, that is, from the mother. He asks for a word that is able to temper and stabilize the imaginary tension, opening toward the future: a sign of consent, of beseeched love—in short, an answer that comes from the other.

It is here that Lacan values what Freud had presented as the second type of identification in chapter VII of *Group Psychology and The Analysis of The Ego*: that is, the constitution of the ideal of the ego as there is suddenly identification in this place from which the subject sees itself as lovable or unlovable, desirable or undesirable. Here, it finds an answer to its question, but how do we call this trait? Lacan speaks here of sign, of image of *a*: The subject internalizes this unary trait as a sign, says Lacan, but can we speak here properly of sign? The objection arises from the fact that we define sign as that which represents something for someone although identification is at stake here. For example, the dog sees signs, but what happens to itself in its representation? It is evident that it is taken in the image, and this is the reason why the human being gets a deep sense of peace from animal company as this excludes the ambiguity of the signifier (Lacan, 1961-1962, [1958-1959]).

This is why Lacan specifies that the trait is not of the order of the mimesis or of the figurative resemblance of the sign but of the order of the pure signifier that—in its dimension of trace, of letter—represents the subject. Therefore, Lacan reports what Freud calls a unary trait (*einziger zug*) to the countable one of the brand, thereby pointing out that no pre-established attribute is involved in it as well as no qualities that would be intrinsic to the name.

From this, it is clear how the heterogeneity of this trait with respect to the imaginary is radical. The ideal of the ego, consisting of unary traits, is a symbolic projection that determines and supports the imaginary projection on the ideal ego because it transcends it Lacan (2006 [1960c]). Some questions remain open: The answer of the other, is it in the order of love or desire? What happens to the imaginary lack as such, i.e., -φ?

In the third stage, there is a new presentation of the mirror stage. This is especially evident in the abovementioned preface *On My Antecedents* written in 1966, where it is said that, if, in the first stage, it was a precise biological cause (i.e., the delay of nerve coordination linked to premature birth) to determine the effort in the child to make up for its present insufficiency anticipating its unity through the image of the other, matrix of the mirror image, whence the motion of jubilation; here, now, Lacan distances himself from this first vision by saying that the lack that causes the assumption of the specular image is not at all a physiological insufficiency.

Also, this would mean supposing a harmony in the animal between Innenwelt and Umwelt, which the child could reach through the imaginary. Even worse would be to let people believe that this harmony would be found in the other and that one could anticipate it through its image.

In the writing *On My Antecedents*, Lacan clearly says that jubilation is not the result of the resolution of an organic fault.

What is manipulated in the triumphal assimilation of the image of the body in the mirror is this object that is the most evanescent that can be imagined and that appears only on the margins; the exchange of gazes manifest in the moment in which the child turns toward the one who somehow assists him, also simply to watch over him while playing.

What matters is not only the child insofar as he is the viewer, but the fact of knowing himself as the object of the other’s gaze as it is expounded in 1964 in *The Seminar of Jacques Lacan, Book XI, The Four Fundamental Concepts of Psychoanalysis*. Therefore, the wager is no longer the mastery of vision, but the scopic object as an object that may be missing in the field of the other. What is this lack? Not of the symbolic lack S(Å), but of a lack of the imaginary -φ. In this empty point, the *object a* can be laid as a gaze. Lacan brings a striking example, the scene of a movie in which a young girl looks at herself naked in the mirror while her hand in a flash crosses the phallic deficiency. Then, the jubilation arises from the intersection of glances that comes to cover the phallic deficiency even if this intersection is punctual and evanescent as it befits the most evanescent object there is, that is, the gaze precisely as an object of the scopic drive.

We have already seen that there is a cleft between the eye, the organ of vision, and the gaze: In the field of the other, where the subject perceives itself as seen as lovable, that is not the point from where it is looked at. This would, therefore, be the nonorganic cause of this last definitive remodulation of the stage of the mirror, that which Lacan synthesizes in the somewhat cryptic formula of an alienation that already situates its desire in the field of the other. The causal well-being is not, therefore, the biological inertia but the phallic deficiency in the mirror image. There is a real reversal of perspective: the imaginary of the hole for the drive that will be to come.

## Discussion

We must grasp the whole novelty of this third presentation of the mirror stage. It tells us that the mere sighting of the image in the other is not enough to constitute the image of one’s own body. In fact, if the image of one’s own body were founded on the basis of the sighting of the image of the other, then we should deduct that the blind would not have ego; instead, the effectiveness of identification comes from the gaze in the field of the other, a gaze to which even the blind is subjected. What is the peculiarity of this imaginary that hinges on the gaze?

The constitution of the mirror image in its first version was inseparable from the seeing quality of the subject. This imagery gave the ego the character of a body surface delimiting a substantial figure full and bounded as it consists of that mirror that is the similar other. On the contrary, if it is the gaze of the other that is placed at the basis of this construct, then it follows that the image of the other is pierced and that the *object a* in the field of the other comes precisely in the place of this hole that is -φ in the image. This is why Lacan can say, in the 1970s, that the ego is a hole and that no underlying substance can be assumed anymore.

By comparison, it is clear why in those same years Lacan can affirm that the *object a* is what makes the image hold or also that, precisely because the image is pierced, it can only stand thanks to *object a*.

All this, obviously, is not imaginable…unless we establish another way of imagining, an imaginary dimension, a new way of naming the imaginary. It is a matter of thinking of the hole not as something that would produce itself retroactively on a totality formed after the laceration of a surface, but on the contrary as something that generates the figure: in short, it is a matter of thinking of the structure of the board as an operator (Lacan, 1971). This new imaginary demands the abandonment of the metric properties of classical geometry for a topology in which the qualitative properties of proximity are maintained even after the transformations: in this way, it is possible to achieve the invariant properties of the figure, that is, they remain so after a continuous transformation without ruptures or overlaps. It is an imaginary no longer linked to narcissism and ego.

We are witnessing a continuous transformation of an image in its inverse starting from -φ. Kant in the *Prolegomeni* (1950, [1783]) had already posed the problem, pointing out that the similarity between a hand or ear and the respective mirror image was only deceptive appearance. In fact, you cannot replace the image of your right hand with the image of your right hand in the mirror because the image of your right hand in the mirror is actually a left hand. The really peculiar thing is that there are no conceivable internal differences with the intellect between the two images, and yet the differences are intrinsic because the left hand can never be bound in the same limits as the right hand despite their similarity. They can in no way coincide and the glove of the left hand cannot serve at all to cover the right hand. Kant wondered what could be the solution to this strange, stumbling block.

It is as if in fact Lacan took up this gauntlet launched by Kant (Lacan, 2016, [1975-76]). Lacan states that there can be no solution if you remain anchored to the intuition intended as eyesight that, through the light beam, hits substance objects. Lacan says that the solution to the problem posed by Kant, staying with the example of the glove, can only be that of overturning the glove: the glove covering the right hand cannot be used to cover the left hand unless it is overturned.

In this way, a right–left inversion can take place, that is, an inversion of naming, thanks to the operation of overturning. This overturning operation can only take place on the condition that the glove be a perforated surface, that is, supplied with an opening.

This imaginary is something different from the one related to narcissism, to which we had been introduced in previous years. This new imaginary is the one suitable to change the relationship of the imaginary with the symbolic and with the real. In this moment, the visual field is, on the one hand, the cardinal principle of the consistency of the experience of reality (as imaginary) although, on the other, it is an element of irreducibility to reality (as object gaze).

In the Lacanian field, there are no studies as intended in terms of academic research because each experience with each patient is unique. One must bear in mind that, generally speaking, nothing works for everyone in psychoanalytic treatment although, at the same time, an analyst with a large experience in the field can give clinical and theoretical testimony. This is the case of Bruce Fink (2007). In his book, he writes about the presence of the gaze in psychoanalytic treatment at a distance via telephone sessions even if the therapist is not physically present. Fink considers this to be a variation of the psychoanalytic situation. What emerges is that, in some patients, all the gaze-related phenomena that we have come to expect from in-person sessions have also arisen in phone sessions. Miller (1999) stated that “in the session, [the analyst and the analysand] are together, synchronized, but they are not there to see each other, as is clear by the use of the couch. The mutual presence in flesh and blood is necessary, if for no other reason than to have emerge that the sexual non-relation.” With the birth of new technologies, new tools are used for the remote psychoanalytic treatment, like Skype and FaceTime. All of these new tools imply a display, a screen in which the patients can see the analyst and, at the same time, they can see themselves. In the gaze on the screen, therefore, the scopic drive circuit is set in motion. In front of the screen, one is, first of all, looked at; one becomes the spectacle of the gaze of the other. We are, Lacan says, (Lacan, 1988, [1964c]) being watched in the spectacle of the world. What makes us aware also establishes us as *speculum mundi*. The smartphone used by the patients or the computer with the intermediation of the screen unleashes a drive circuit in which being looked at and looking intertwine, intersect. In our opinion, the circulation of the scopic drive that occurs in front of the screen can be represented through the overturning of a torus by perforation, which causes the overturning of the inside and the outside: that is, what is internal becomes external and vice versa. In our opinion, this is certainly a new way in which the functioning of the gaze object is active in psychoanalytic treatment in the Lacanian field. To organize and understand how this new way of functioning of the gaze impacts the process of psychoanalytic treatment, the transition it produces, this aspect needs to be studied further. Case studies could thereby be of key significance.

## Author Contributions

CL is the main contributor of this manuscript. CA, AS, and DC contributed to its conception and revised critically the manuscript for language and intellectual content. All authors contributed to the article and approved the submitted version.

## Conflict of Interest

The authors declare that the research was conducted in the absence of any commercial or financial relationships that could be construed as a potential conflict of interest.
